# Meropenem-Resistant Achromobacter xylosoxidans, Subspecies Denitrificans Bacteremia in a Patient With Stage IV Adenocarcinoma of the Lung

**DOI:** 10.7759/cureus.15546

**Published:** 2021-06-09

**Authors:** Andre M Agassi, Erin Pollock, Mary M Carter, Robert J Sherertz, Andrew P Mangano

**Affiliations:** 1 Department of Internal Medicine, Grand Strand Medical Center, Myrtle Beach, USA

**Keywords:** meropenem, achromobacter xylosoxidans, neutropenic enterocolitis, clinical case report, immunocompromised hosts

## Abstract

*Achromobacter xylosoxidans*,subspecie*s denitrificans *is a rare Gram-negative bacillus that causes health care associated infections in immunocompromised hosts. Carbapenems and anti-pseudomonal penicillins are listed as suitable empiric therapy in the literature. Herein, we report a case of a 77-year-old male with stage IV adenocarcinoma of the lung who presented with and was improving from *Salmonella javiana* enterocolitis, only to subsequently develop *A. xylosoxidans*,subspecies* denitrificans *bacteremia that was resistant to both meropenem and piperacillin-tazobactam. With empiric antibiotic coverage falling short of microbial clearance, timelyin vitrosusceptibility testing and prompt infectious disease consultation are of the utmost importance for treatment.

## Introduction

*Achromobacter xylosoxidans*, subspecies *denitrificans* is an aerobic, motile, non-fermenting Gram-negative bacillus, commonly found in wet environments (soil, water, gastrointestinal tracts) that mainly causes healthcare-associated infections [1,2]. There are relatively few cases of infection reported in the literature. Of those reported, the majority of cases are in immunocompromised or structurally damaged hosts [3]. A frequent link with cystic fibrosis is of particular clinical relevance, as outbreaks have been known to occur [2,4,5]. The treatment of *A. xylosoxidans *can be difficult as the microorganism carries both intrinsic and acquired mechanisms of resistance, often conferring a phenotype of multidrug resistance (MDR) [6-8]. Carbapenem therapy is commonly recommended in case reports, because meropenem resistance has been infrequently noted in previous studies [9-11]. We report a case of *Salmonella javiana* enterocolitis with subsequent development of *A. xylosoxidans*, subspecies *denitrificans* bacteremia that is resistant to both meropenem and piperacillin-tazobactam.

## Case presentation

A 77-year-old male with a past medical history of chronic obstructive pulmonary disease (COPD), coronary artery disease (CAD), and stage IV adenocarcinoma of the lung, with metastatic disease to the brain and bone, presented to the hospital with severe mouth pain and odynophagia in the Fall of 2020 in the Southeastern United States. The patient had just completed his second round of chemotherapy 10 days prior to admission. The patient’s chemotherapy regimen consisted of gemcitabine, paclitaxel, and bevacizumab. The patient’s home medications included clopidogrel, clotrimazole, doxycycline, enoxaparin, fluconazole, as well as dexamethasone for control of metastatic brain lesions. Initial vital signs revealed a pulse rate of 117, a respiratory rate of 18 breaths per minute, an oxygen saturation of 94% on room air, and a blood pressure of 78/52 mmHg. Physical examination noted a chronically ill-appearing male with severe stomatitis, dry mucous membranes, a white plaque on the tongue, tenderness to palpation in the lower abdominal quadrants, and 2+ pitting edema in bilateral lower extremities. Initial laboratory analysis revealed a white blood cell count of 0.5 K/mm^3^ (absolute neutrophil count of 90 cells/µL), a hemoglobin of 13.2 gm/dL, and a platelet count of 76 K/mm^3^. Blood glucose was elevated to 425 mg/dL. Blood cultures were obtained prior to initiating antibiotics.

**Figure 1:**
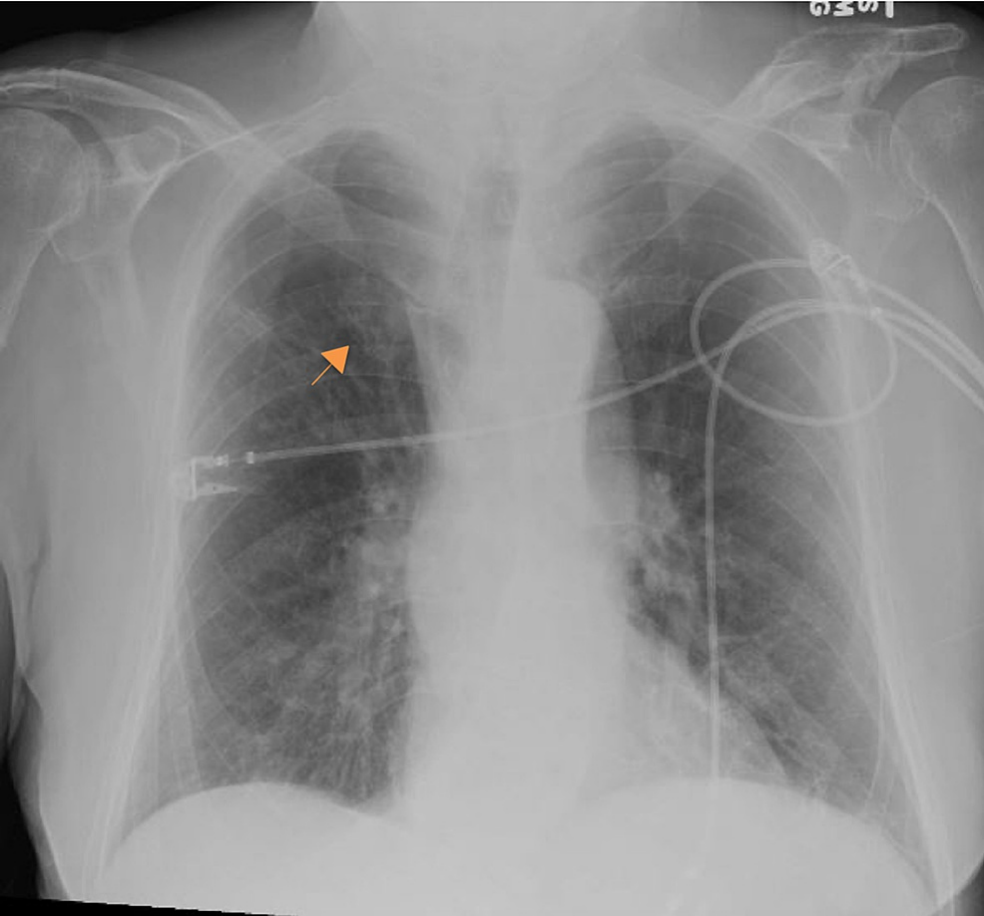
Portable chest film showing a diffuse coarsened interstitial pattern and a right upper lobe density (orange arrow)

**Figure 2:**
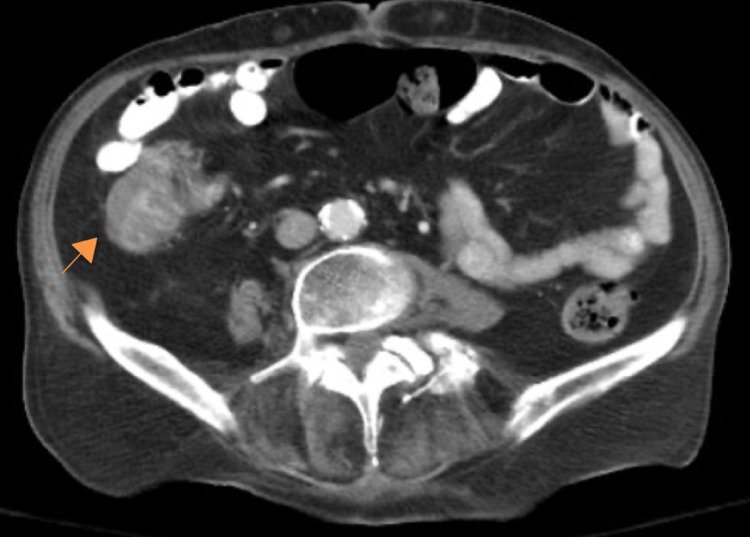
Axial CT image, with both IV and PO contrast, demonstrating thickened inflammatory changes in the ascending colon (orange arrow)

Portable chest film (Figure 1) demonstrated a small density in the right upper lobe with a coarsened interstitial pattern overall. A questionable nodularity was noted in the right infrahilar region. These findings were largely unchanged from previous films. A CT abdomen and pelvis with contrast (Figure 2) noted inflammatory changes and thickening in the ascending colon extending into the transverse colon.

The patient was admitted with a presumptive diagnosis of neutropenic enterocolitis and was treated with Meropenem (initially at a dose of 1 g every eight hours), fluid resuscitation, and a subcutaneous insulin regimen. After discussing the case with the patient’s oncologist, filgrastim (300 mcg) was given to improve the patient’s neutropenia on hospital day three. Admission blood cultures remained negative, and the patient slowly improved. On hospital day 4, the patient developed diarrhea. Subsequently, stool polymerase chain reaction (PCR) and *Clostridium difficile* assays were obtained. *C. difficile *assay was negative, but stool PCR detected *Salmonella javiana*, the likely cause of his enterocolitis. The patient continued to improve, and another dose of filgrastim (300 mcg) was given on hospital day 6 due to continued neutropenia.

On hospital day 7, the patient developed hypotension to 85/51 mm/Hg, respiratory distress, and metabolic acidosis. Now, meeting requirements for septic shock, he was transferred to the intensive care unit (ICU) due to a new norepinephrine requirement. The abdominal X-ray did not show any acute pathology and chest X-ray findings were unchanged. Repeat blood cultures were obtained. Within 24 hours, Gram-negative bacilli grew on the repeat blood cultures. The patient continued to decline with the development of acute renal failure, worsening encephalopathy, and respiratory failure. On hospital day 10, he was transitioned to comfort care, given his advanced cancer and poor response to therapy. After his death, the cultures resulted; all four cultures initially obtained in the ICU were growing a multidrug-resistant *Achromobacter xylosoxidans*, subspecies* denitrificans *strain. The microbiological sensitivity analysis is outlined in Table 1.

**Table 1:** Microbiological sensitivities of the A. xylosoxidans species isolated

Antibacterial agent	Sensitivity
Cefepime	Resistant
Ciprofloxacin	Intermediate
Gentamicin	Intermediate
Levofloxacin	Sensitive
Meropenem	Resistant
Piperacillin tazobactam	Resistant
Tetracycline	Intermediate
Tobramycin	Intermediate
Trimethoprim/sulfamethoxazole	Sensitive

## Discussion

*A. xylosoxidans* is thought to be a low virulence pathogen, occurring mainly in nosocomial settings. Infection tends to occur in immunocompromised individuals, though cases in immunocompetent patients have been documented previously [9,12]. In a ten-year analysis of 52 cases in 46 patients, 24 patients (52%) had neutropenia (<500 cell/µL), and of the 52 cases, 13 (25%) were associated with an infected intravascular catheter [13]. Our patient was indeed immunocompromised with neutropenia following chemotherapy, but a central venous line was not placed until transfer to the ICU. Since *S. javiana* and *A. xylosoxidans* both can stem from environmental sources, it is quite possible they originated from the same environmental source in this case. The patient survived the Salmonella infection because the organism was likely sensitive to meropenem (no sensitivities available), but did not survive the Achromobacter infection due to antibiotic resistance.

Based on a review of two large case series, one with 54 cases and the other with 52 cases, *A. xylosoxidans *are often susceptible to carbapenems and anti-pseudomonal penicillins but resistant to second-and third-generation cephalosporins as well as gentamicin. Results from the two studies revealed variable susceptibility in regards to ampicillin-sulbactam and fluoroquinolones. Curiously, the studies were split on the usage of trimethoprim/sulfamethoxazole (TMP-SMZ), with one study stating that most isolates were resistant, and the other stating that most isolates were susceptible [10,13]. Antimicrobial combinations have been used for treating* A. xylosoxidans *infections previously, though predicting a suitable multidrug regimen would be considerably difficult. Such combinations as azithromycin plus TMP-SMZ or azithromycin plus doxycycline have been documented previously [11]. 

Our patient’s strain, as seen above, was resistant to cefepime, meropenem, and piperacillin-tazobactam. This is a concerning resistance pattern as meropenem and piperacillin-tazobactam are frequently selected as empiric agents until cultures result in practice. The strain was intermediate to a number of antibiotics including gentamicin and tetracycline. The term intermediate is conferred when the sensitivity of a bacterial strain to a given antibiotic is inhibited in vitro by a concentration of the drug that is associated with an uncertain therapeutic effect [14]. Intermediate susceptibility is far from ideal antibiotic coverage with much higher than normal dosages of antibiotic being required to treat the organism. Our patient’s strain was susceptible to only two antibiotics tested, levofloxacin and TMP-SMZ. As stated above, fluoroquinolones and TMP-SMZ were not noted as first-line therapies in two large case series of *A. xylosoxidans* infections.

In reviewing the medical record, this patient had no previous carbapenem exposure. Given the patient's treatment with meropenem during his initial Salmonellainfection, it is likely this resistant *A. xylosoxidans* strain was induced during his hospital course. Carbapenem resistance is a growing public health threat, particularly in Gram-negative bacteria in which mechanisms of drug resistance can be numerous [15,16]. When a patient develops sepsis while already on a carbapenem, it would be wise to consult an infectious disease specialist, as empiric treatment in this situation could likely require a combination therapy and include a non-first line agent, such as polymyxin.

## Conclusions

Advanced systemic infections caused by multidrug-resistant bacteria continue to be a growing concern, especially in high-risk individuals. Infection due to *A. xylosoxidans *can be life-threatening in neutropenic patients with cancer-related immunosuppression. Despite *A. xylosoxidans* reported susceptibility to carbapenems and anti-pseudomonal penicillins, these empiric agents alone may not be appropriate in a subset of patients. If promptly reported, antibiotic coverage should be altered based on available in vitro susceptibility testing. Future studies on this rare Gram-negative bacillus are needed to further discern ideal initial therapy. Antibiotic combinations, guided by an infectious disease specialist, may be a more prudent treatment rather than a single reserved antibiotic alone in patients with refractory sepsis. This is especially true in patients whose microbiological sensitivity patterns are not yet available.
